# Potential Anticancer Effect of *Cannabis sativa* L. Dichloromethane Extract Through Oxidative Stress-Related Pathways and the Inhibition of the Migration and Invasiveness of Human Breast Cancer Cells (MDA-MB-231 and MCF-7)

**DOI:** 10.3390/ijms27010152

**Published:** 2025-12-23

**Authors:** Corinne Raïssa Ngnameko, Jacqueline Njikam Manjia, Motlalepula Gilbert Matsabisa

**Affiliations:** 1 African Medicines Innovations and Technologies Development, Department of Pharmacology, Faculty of Health Sciences, University of the Free State, P.O. Box 339, Bloemfontein 9300, South Africa; corinnengnameko@yahoo.fr (C.R.N.); jmanjia@gmail.com (J.N.M.); 2 Pharmacology and Drug Discovery Laboratory, Institute of Medical Research and Medicinal Plants Studies, Yaoundé P.O. Box 13033, Cameroon; 3 Laboratory of Pharmacology and Toxicology, Department of Biochemistry, Faculty of Science, University of Yaoundé I, Yaoundé P.O. Box 812, Cameroon

**Keywords:** *Cannabis sativa* extract, breast cancer, apoptosis, pro-oxidants, metastasis

## Abstract

Breast cancer remains a leading cause of cancer-related morbidity and mortality globally, highlighting the urgent need for novel therapeutic strategies. This study investigates the molecular mechanisms underlying the anti-proliferative potential of *Cannabis sativa* dichloromethane extract (*C. sativa* DCM) on oxidative stress, apoptosis, and invasion in human breast cancer cells. Key biomarkers, such as antioxidant enzymes (Superoxide Dismutase (SOD) and Glutathione (GSH)), the transcription factor Nrf2, apoptotic proteins (p53, caspase-8 and 9), metalloproteinase (MMP-1 and MMP-9), and Transforming Growth Factor Beta (TGF-β) were examined. Cytotoxicity was assessed using an MTT assay in the MDA-MB-231 and MCF-7 breast cancer cell lines, with comparisons to normal skin fibroblasts (HS27). Oxidative stress biomarkers were quantified using enzymatic assays and ELISA kits, while apoptotic and anti-metastatic factors were determined by Western blotting. Results demonstrated that *C. sativa* DCM extract induced significant cell death in a concentration-dependent manner, with IC50 values of 75.46 ± 0.132 μg/mL for MDA-MB-231 and 78.68 ± 0.50 μg/mL for MCF-7 cells. The extract decreased SOD and GSH levels while increasing p53 and caspase activity, confirming apoptosis activation. Additionally, *C. sativa* DCM inhibited migration and invasion by downregulating MMP-1, MMP-9, and TGF-β. The anti-proliferative potential of *C. sativa* DCM in breast cancer cells is mediated through a continuous biological pathway involving oxidative stress modulation, apoptotic signaling, and anti-invasive effects. Phytochemical analysis revealed terpenoids and steroids, including compounds like cannabidiol and tetrahydrocannabinol acid. These findings suggest that *C. sativa* DCM extract holds potential as an anti-breast cancer therapeutic and warrants further preclinical and clinical investigations.

## 1. Introduction

Cancer remains one of the most important health problems, with increasing mortality rates. According to the World Health Organization (WHO), the global incidence (in 2022) was estimated at 20 million new cases, up to 9.7 million deaths, and it is expected to reach 35 million in 2050, making cancer the second-leading cause of death, particularly affecting low- and middle-income countries [1]. To date, breasts are more susceptible to developing cancer than other organs, and breast cancer becomes the second leading cause of death of women worldwide, with a global prevalence of 2.3 million new cases recorded in 2022 [1].

The exact mechanism underlying breast cancer is still not well understood; however, significant research has focused on cellular processes and pathways involved in the development of breast cancer, such as cell survival, proliferation, migration, differentiation, oxidative stress, angiogenesis, and apoptosis. These signal transduction pathways frequently cross-talk with each other to ensure breast cells respond appropriately to the extracellular growth factors [2,3]. An imbalance in antioxidant defense could be responsible for mitochondria-oriented apoptosis [4,5,6]. Apoptosis is a crucial physiological and biological process for normal development and homeostasis maintenance, primarily regulated by the activation of a class of cysteine proteases called caspases [7,8].

Reactive oxygen species (ROS) are normally generated during metabolic processes, and in healthy humans, the concentration of these dangerous molecules is controlled by cellular antioxidant enzymes such as SOD, catalase (CAT), glutathione peroxidase (GPx), GSH, glutathione reductase (GR), and antioxidant molecules [9,10]. Both non-enzymatic and enzymatic antioxidant systems protect the cells by neutralizing the harmful effects of ROS. Nevertheless, controversial issues concerning the interference between chemotherapy, ROS, and antioxidants need to be understood to enhance combined therapies [11,12,13]. Within this context, numerous compounds have been reported to influence intracellular levels of free radicals associated with carcinogenesis. Some of these compounds act as pro-oxidants, while others act as inducers of antioxidant enzymes, modulating oxidative stress and impacting breast cancer progression [14,15,16].

Matrix metalloproteinases (MMPs) are another group of enzymes that belong to a family of extracellular endopeptidases that selectively degrade components of the extracellular matrix. These enzymes may be involved in endothelial cell attachment to the extracellular matrix, their detachment, and their migration/invasion, which is important in cancer treatment. These enzymes are produced by various cell types, including epithelial cells, fibroblasts, and inflammatory cells. Some of the MMPs produced by endothelial cells include MMP-1, MMP-2, MMP-9, and MT-1-MMP [17,18].

Interest in phytotherapeutic drugs is growing due to their potential to be used alongside other treatments and dietary supplements during recovery, helping to prevent tumor recurrence and drug resistance while minimizing side effects from primary therapies [19,20,21]. Although cancer treatment is making significant progress, it often involves expensive procedures and medications, with chemotherapy’s side effects causing considerable patient suffering. Therefore, new approaches to find effective, better-acting, and less harmful anticancer drugs are highly sought after. Phytopharmaceuticals, rich in pro-oxidant, anti-inflammatory, and anticancer characteristics, present a promising solution for cancer treatment [22,23,24]. Oxidative stress, characterized by an imbalance between reactive oxygen species (ROS) and detoxification, can promote the progression of breast cancer. Plant extracts modulate this stress by enhancing the antioxidant response and activating the Nrf2 pathway, which stimulates detoxification enzymes and promotes apoptosis. High oxidative stress activates the p53 protein and caspases, inducing cell death. Furthermore, plant extracts can inhibit matrix metalloproteinases (MMPs), thereby reducing cancer cell invasion and establishing a link between oxidative stress and metastatic potential [25,26,27].

*Cannabis sativa* L., known in many slang languages as marijuana, bhang, ganja, for instance, is an herbaceous species originating from Central Asia and widely distributed around the world. It has been used as a source of fiber, food, oil, and for its multiple curative properties, including anti-parasitic, antipyretic, antibacterial, antitumor, vermifuge, dermatic, and pain-killing properties for centuries [28,29]. Phytocannabinoids, derived from *cannabis*, have shown anti-cancer activity in cell lines; however, very few studies have examined the effectiveness of this cannabis extract against breast cancer [30,31]. Recent studies have shown that a dichloromethane extract of South African *C. sativa* L. landrace exhibits inhibitory effect on breast cancer (MCF-7) cell growth and angiogenesis due to its ability to inhibit nitric oxide (NO) and vascular endothelial growth factor (VEGF) [32]. These results suggest a need for further investigation of these extracts from South African *C. sativa* into the mechanism of action of this extract, particularly regarding its effects on metastasis, oxidative stress, and apoptotic activity in breast cancer cells.

## 2. Results and Discussion

### 2.1. Assessment of Cell Viability

**Figure 1:**
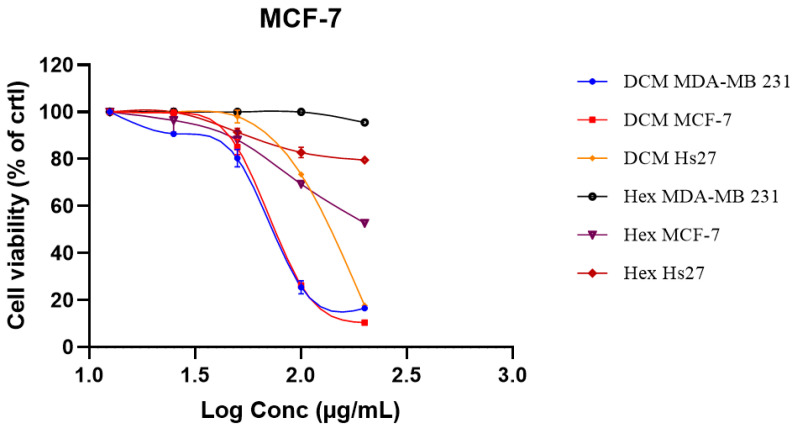
Cytotoxic effect of *Cannabis sativa* extracts on MCF-7, MDA-MB-231, and Hs27 cells. Ten thousand cells were seeded per well for each cell line on 96-well microliter plates, and the cells were treated for 24 h with different extracts (12.5–200 μg/mL) under standard cell-culture conditions. The cell viability was estimated as a percentage of cells treated with DMSO (0.6%), considered as 100%. Values are means ± SD of three independent experiments in triplicate. Hex—hexane extract; DCM—dichloromethane extract.

*C. sativa* has gained a lot of attention due to its pharmacological properties, including anti-parasitic, antipyretic, anti-bacterial, and antitumor effects [28,29]. According to some studies, Phytocannabinoids, derived from *cannabis*, have shown anti-cancer activity in cell lines [30,31]. Notably, *C. sativa* has demonstrated cytotoxic effects against several cancer types, such as breast, gastric, prostate, lung, liver, oral, and colon cancer cells [33,34,35,36,37]. This present study found that *C. sativa* had a significant anti-cancer effect on MCF-7 and MDA-MB-231 cells by suppressing cell viability in a concentration-dependent manner (Figure 1). The DCM crude extract was obtained after sequential maceration. After the extraction of the plant material, cell viability was estimated as a percentage of DMSO-treated cells (0.6%). The bioassay investigation of *Cannabis sativa* extract was performed on cancer cell lines, including MDA-MB-231 and MCF-7, as well as non-cancerous Hs27 cells, using an MTT assay. The extract was tested from 12.5 to 200 μg/mL. Results showed an inhibition of cell viability in the tested cancer cell lines at different concentrations. The inhibitory concentrations (IC_50_) of each extract can be observed in Table 1.

This table shows that the hexane extract is not effective against all cell lines up to 200 μg/mL. Moreover, as can be seen in Figure 1, the DCM extract demonstrated a reduction in cell viability at the higher concentration of 200 μg/mL after 24 h (Figure 1). Furthermore, dichloromethane extract exerted a similar cytotoxic effect against both cancer cells, with an IC_50_ of 75.46 μg/mL and 78.68 μg/mL observed on MDA-MB-231 and MCF-7 cells, respectively. The extracts did not show any toxicity on normal Hs27 cells up to the concentration of 200 μg/mL, which allowed for the calculation of the selectivity index of these extracts. The present findings are aligned with previous studies indicating that *C. sativa* inhibits the proliferation of MCF-7 cells in a time and concentration-dependent manner [32]. Importantly, *C. sativa* exhibited minimal effect on Hs27 normal cells (normal skin fibroblast cells), which highlights its selective cytotoxicity.

The selectivity index (SI) is a reliable parameter that determines the ability of a given plant extract or compound to inhibit the growth of malignant cells while preserving healthy and normal cells [38]. From this study, it is shown that the ability of the *C. sativa* DCM extracts to inhibit cancer cells could be due to the presence of potent photochemical compounds that selectively target specific functions of malignant cells, thus inhibiting their growth and survival. An SI greater than 1 indicates a greater cytotoxic effect against cancer cells than against normal cells [38]. Based on the SI results, the focus for this study was on DCM (dichloromethane) extracts (see Table 2). Moreover, *C. sativa* not only inhibited the proliferation but also induced apoptosis in MDA-MB-231 cells, suggesting its potential as a targeted therapeutic agent for breast cancer treatment with reduced toxicity on healthy tissues.

### 2.2. Cannabis sativa Dichloromethane Extract Inhibited Colony Formation of MCF-7 and MDA-MB-231 Cells

**Figure 2:**
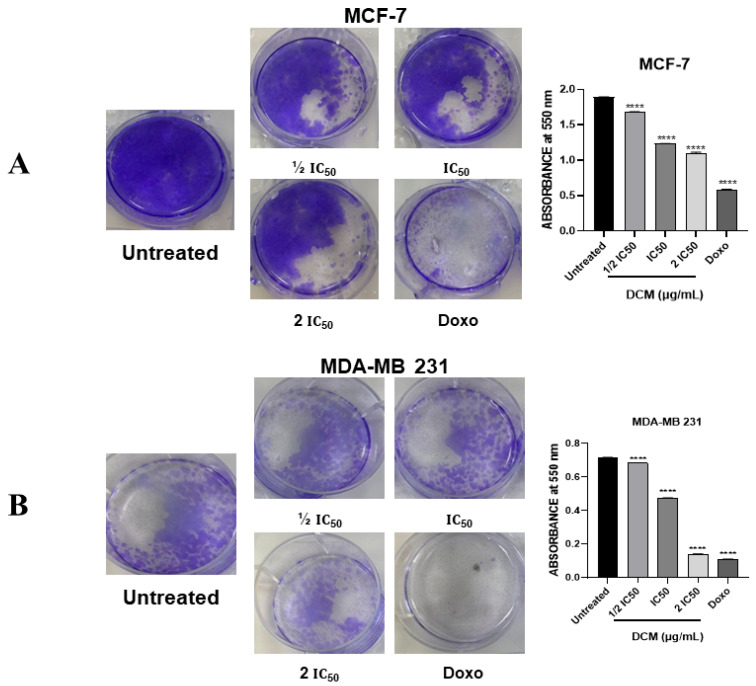
Colony formation after treatment with *C. sativa* dichloromethane extract. One thousand cells from cell lines MCF-7 (**A**) and MDA-MB-231 (**B**) were seeded in 6-well microliter plates and exposed to *C. sativa* DCM for 24h, followed by the replacement of the culture medium every 3 days for 15 days. The colonies were stained with crystal violet (0.5%) and allowed to dry overnight. Then, 2 mL of acetic acid (33%) was added per well, and the absorbance was read at 550 nm using a spectrophotometer. DMSO was used as a negative control (untreated). Values are means ± SD of three independent experiments. Differences among the treated and vehicle-treated control groups were determined by one-way ANOVA. **** *p* < 0.0001, as compared to untreated group. DCM—dichloromethane extract.

To assess the capacity of single cells to survive brief exposure to the test agent, cannabis DCM extract, while maintaining their ability to proliferate, clonogenic assays were performed. The results indicated that the colony-forming ability of the cells was significantly reduced with increasing concentrations of *C. sativa* DCM after 24 h of treatment (Figure 2). Notably, a remarkable inhibition of colony formation was observed with the DCM extract at 2 × IC_50_ on both cancer cell lines, with fewer colonies formed as compared to untreated cells. This significant reduction in the formation of colonies indicated that after exposure to DCM extract, the damage caused to both cancer cells was irreversible in such a way that these treated cells were unable to grow again after 15 days. Therefore, these data confirmed the antiproliferative potential of DCM extract against both cancer cells. The treated cells lost their ability to proliferate and form colonies due to persistent DNA damage or the disruption of critical cellular processes, which could not be repaired. Thus, the colony suppression indicated long-term proliferative failure rather than acute cytotoxicity, reflecting a sustained impact on the cell’s reproductive integrity, thereby leading to an inability to regenerate a viable population [39,40,41].

### 2.3. Cannabis sativa Inhibited the Migration of MDA-MB-231 and MCF-7 Cells

**Figure 3:**
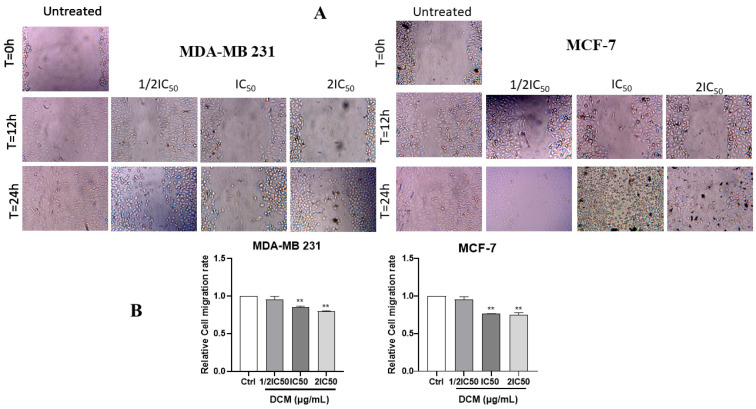
Microscopical images representing the in vitro cell migration of MDA-MB-231 and MCF-7 cells incubated in the presence or absence of the *C. sativa*. Images were captured at 0, 12, and 24 h, and quantitative closure (%) for the scratched assay. (**A**) Representative images of the scraped area; (**B**) quantification of the migrated area in relation to the scraped area. Results are expressed as the percentage of scraped area. DMSO was used as a negative control (Ctrl). Values are means ± SD of three independent experiments. Statistical differences were determined by one-way ANOVA. ** *p* < 0.01, as compared to the untreated group. DCM—dichloromethane extract.

The effect of *C. sativa* on MDA-MB-231 and MCF-7 cell migration was evaluated. As displayed in Figure 3A,B, the treatment with dichloromethane *C. sativa* extract significantly reduced the migration of the breast cancer cells. These results demonstrate that DCM extract inhibited the migration of MDA-MB-231 and MCF-7 cells in a concentration-dependent manner.

In agreement with the previous study, these findings reveal that *C. sativa* DCM at the tested concentrations can increase the proportion of total apoptotic cells in MDA-MB-231 cells.

### 2.4. Effect of Cannabis sativa Extracts on Metalloproteinase (MMP-1 and MMP-9) and Transforming Growth Factor (TGF) Beta 1

Matrix metalloproteinases (MMPs) constitute a large family of zinc-dependent proteases, produced in an inactive form and activated through the removal of N-terminal propeptides [42]. High expression of MMP-9, often associated with MMP-2, is generally linked to enhancing the invasiveness of cancers, including breast cancer. MMP-1 was initially identified as an activator of MMP-2 [43]. These MMPs promoted tumor progression and metastasis in invasive cancers by degrading the extracellular matrix (ECM), which consisted of two primary components: basement membranes and interstitial connective tissue. ECM degradation not only promoted the migration of metastatic cancer cells, but also promoted tumor growth by creating space for expansion [44,45,46,47]. Therefore, targeting metalloproteinases represented a promising avenue for breast cancer treatment. In this regard, the effect of *C. sativa* extract on MCF-7 and MDA-MB-231 cells using ELISA kits was explored.

**Figure 4:**
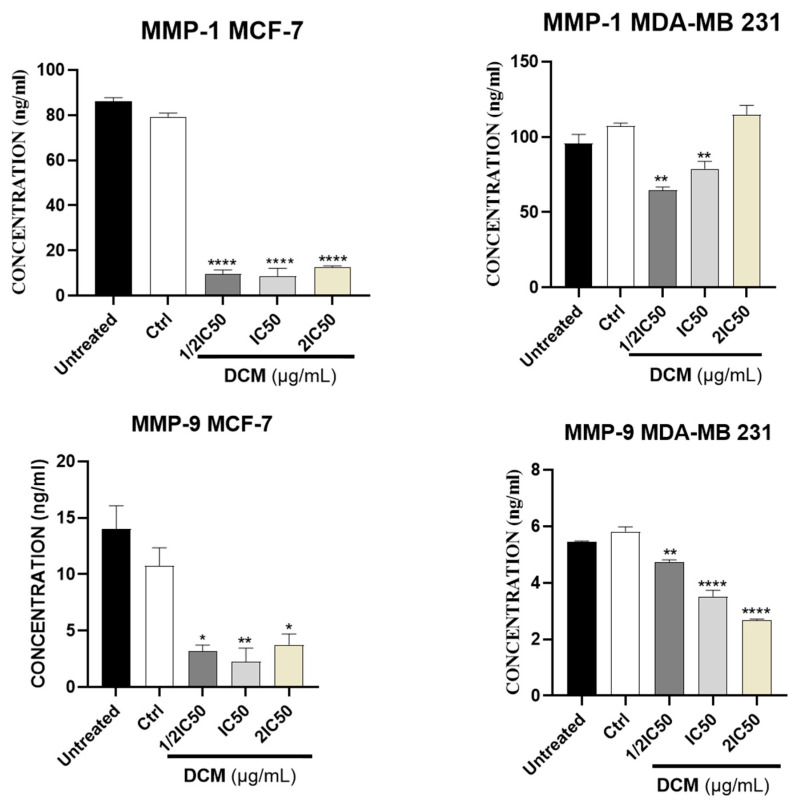
Effect of *Cannabis sativa* on MMP-1 and MMP-9 expression levels in MCF-7 and MDA-MB-231 cells, compared with the negative control. DMSO was used as a negative control (Ctrl). Values are means ± SD of three independent experiments. Differences among the treated and vehicle-treated control groups were determined by one-way ANOVA. * *p* < 0.05, ** *p* < 0.01, **** *p* < 0.0001 against control group. DCM—dichloromethane extract.

**Figure 5:**
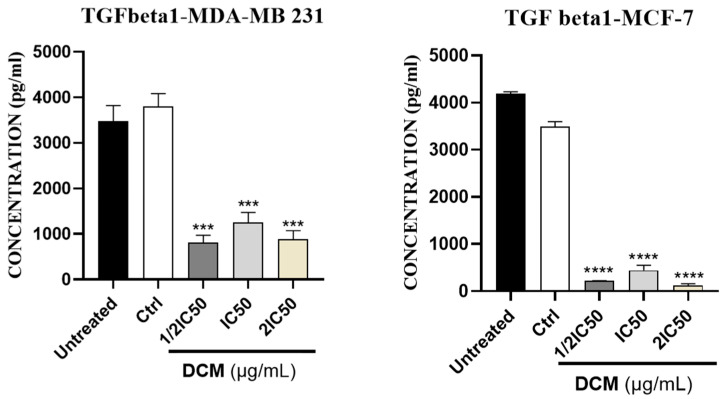
Effect of *Cannabis sativa* on TGF-beta expression levels in MCF-7 and MDA-MB-231 cells, compared with the negative control. DMSO was used as a negative control (Ctrl). Values are means ± SD of three independent experiments. Statistical differences were determined by one-way ANOVA. *** *p* < 0.001, **** *p* < 0.0001, as compared to control group. DCM—dichloromethane extract.

As shown in Figure 4 and Figure 5, our results showed that DCM extract inhibited a statistically significant (*p* < 0.05) amount of MMP-1, MMP-9, and TGF-β protein expression in MCF-7 cells, and in MDA-MB-231, it inhibited the expression of MMP-9 and TGF-β protein. The reduction in MMP-1 and MMP-9, alongside TGF-β modulation, directly influenced functional metastatic dynamics by interrupting the pathways essential for matrix remodeling and cell invasion. MMP-1 and MMP-9 are critical for degrading extracellular matrix components, facilitating tissue invasion by cancer cells. Additionally, TGF-β is involved in epithelial-to-mesenchymal transition (EMT) and promotes a pro-invasive microenvironment. By reducing the expression and activity of these molecules, the *C. sativa* DCM diminished the invasive potential of cancer cells, effectively impairing their ability to migrate through the stroma and establish secondary tumors [48,49,50].

### 2.5. Effect of Cannabis Sativa Extracts on SOD and GSH Concentrations

**Figure 6:**
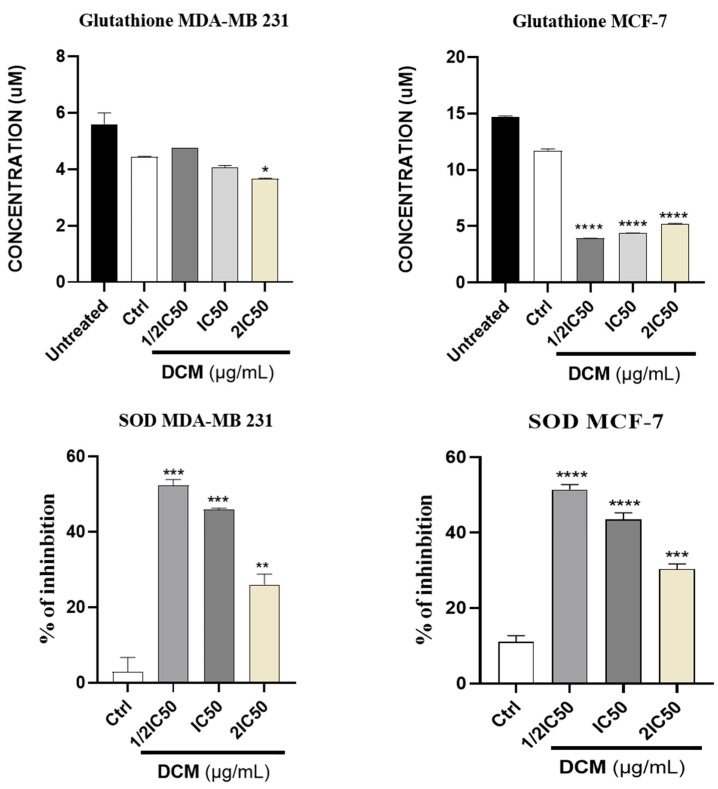
Effect of *Cannabis sativa* extracts on SOD activity and GSH levels on MDA-MB-231 &MCF-7. DMSO was used as a negative control (Ctrl). Values are means ± SD of three independent experiments. Differences among the treated and vehicle-treated control groups were determined by one-way ANOVA. * *p* < 0.05, ** *p* < 0.01, *** *p* < 0.001, **** *p* < 0.0001, as compared to control group. DCM—Dichloromethane extract.

One of the main findings of the study was the modulating of antioxidant activity by *C. sativa* DCM, especially regarding superoxide dismutase (SOD) and glutathione (GSH) levels, in breast cancer cells. Pro-oxidants play a crucial role in increasing reactive oxygen species (ROS), which are known to contribute to cancer progression by inducing cellular damage and promoting cancer cell death [51,52]. This is consistent with previous research indicating that cannabinoids can influence pro-oxidant pathways, particularly the Nrf2 pathway, which regulates the expression of genes involved in pro-oxidant defense mechanisms [13,53,54]. The data showed that *C. sativa* DCM reduced SOD activity and GSH levels in cells treated with different concentrations (Figure 6). Reduced activity of antioxidant enzymes like Superoxide Dismutase (SOD) and Glutathione (GSH) levels leads to an accumulation of reactive oxygen species (ROS), causing oxidative stress. This increase in ROS can overwhelm cellular defenses, altering redox homeostasis and damaging key biomolecules, including DNA and proteins. When the oxidative damage surpasses a specific threshold, it activates pro-apoptotic pathways, such as the activation of p53, which triggers caspase cascades (e.g., caspase-8 and 9). Ultimately, this sequence of events ushers the cell toward programmed cell death (apoptosis), resulting in the elimination of damaged cells and maintaining tissue homeostasis [14,55,56].

### 2.6. Effect of Cannabis sativa Extracts on Nuclear Factor Erythroid 2-Related Factor 2 (Nrf2)

The study also examined the impact of *Cannabis sativa* extract on Nrf2 levels. Elevation of Nrf2 levels has been shown in clinical studies in cancer such as lung, ovarian, melanoma, colorectal cancer, endometrial carcinoma, breast cancer, kidney cancer, pancreatic cancer, endometrial carcinoma, and hepatocellular carcinoma. Moreover, an increase in Nrf2 levels has been associated with therapeutic resistance and metastatic invasion in cancer cells [57].

**Figure 7:**
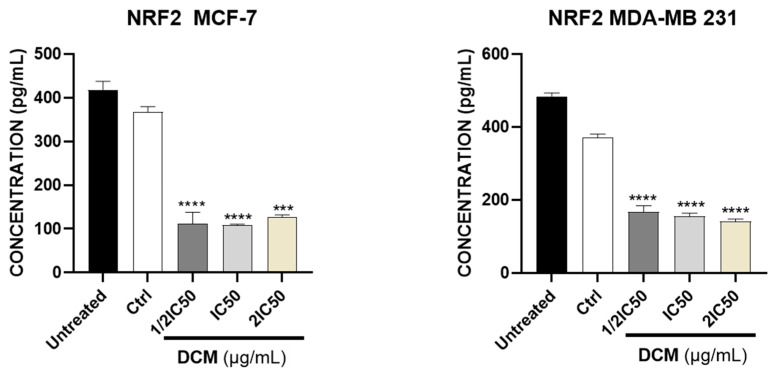
Effect of *Cannabis sativa* extracts on Nrf2 activity on MDA-MB-231 & MCF-7. DMSO was used as a negative control (Ctrl). Values are means ± SD of three independent experiments. Differences among the treated and vehicle-treated control groups were determined by one-way ANOVA. *** *p* < 0.001, **** *p* < 0.0001, as compared to control group. DCM—dichloromethane extract.

These and other findings have suggested that targeting the Nrf2 pathway may be a new cancer therapy. The role of phenolic compounds as anticancer agents due to their inhibitory action on Nrf2 presents new possibilities for new drugs. Apoptosis, or programmed cell death, is a process essential for the elimination of damaged or cancerous cells. Figure 7 shows that *C. sativa* DCM reduces the activity of Nrf2 in both cell lines. These findings suggest a potential mechanism through which *C. sativa* DCM may influence oxidative stress and contribute to its anti-cancer effects. Nrf2 suppression reduces the cell’s ability to activate its antioxidant defenses, leading to an imbalance in intracellular redox potential. This decreased Nrf2 activity induces an increase in reactive oxygen species (ROS), bringing the cell closer to a state of oxidative stress. Consequently, this heightened oxidative environment promotes susceptibility to apoptosis, as damaged cellular components accumulate and activate pro-apoptotic signaling pathways [58,59,60].

### 2.7. Effect of Cannabis sativa Extracts on Caspase-8, Caspase-9, and p53

**Figure 8:**
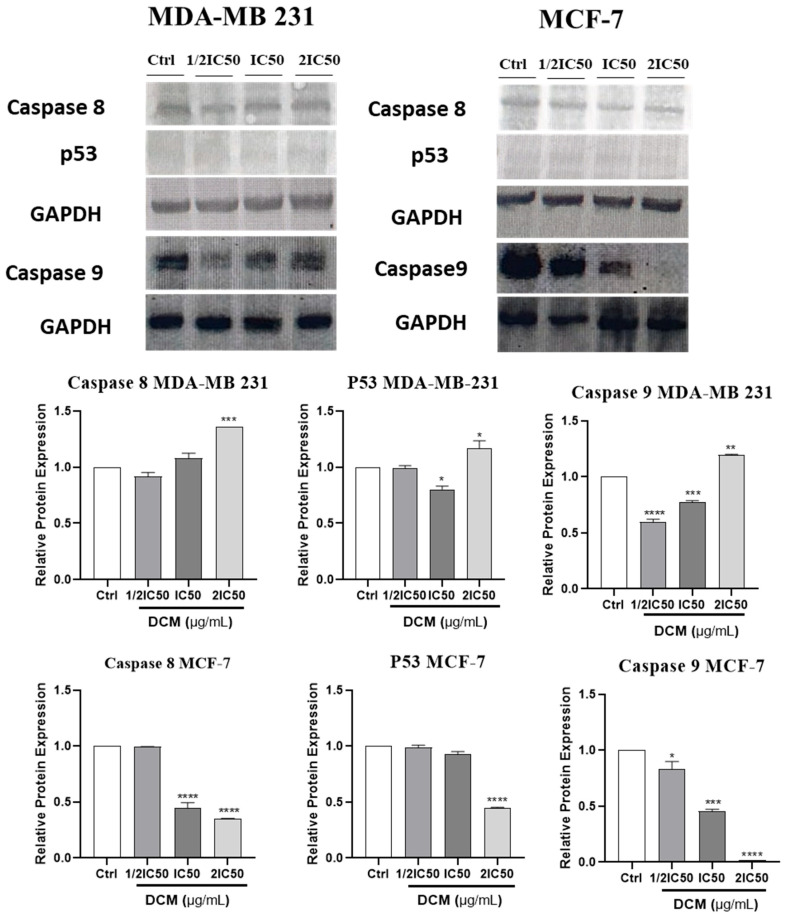
Effect of *Cannabis sativa* on the expression of Caspase-8, Caspase-9, and p53 protein in MCF-7 and MDA-MB-231 cells, compared with the negative control. Cells were treated with various concentrations of plant extract for 24 h, and protein levels of caspase-8, caspase-9, and p53 were determined. After treatment, total proteins were extracted, and caspase-8 and p53 expression were determined by Western blot, with 0.6% DMSO used as a loading control. Each blot represents one of three independent experiments. Values are means ± SD of three independent experiments. Statistical differences were determined by one-way ANOVA, with * *p* < 0.05, ** *p* < 0.01, *** *p* < 0.001, **** *p* < 0.0001 against the untreated control. DCM—dichloromethane extract.

To gain insight into the molecular mechanisms that control cell apoptosis, the action of *C. sativa* DCM on anti-apoptotic p53, and caspase-8 and -9 was investigated. The *C. sativa* DCM induced the expression of the pro-apoptotic protein (Caspase-8, Caspase-9, and p53) in MDA-MB-231 cells (Figure 8). p53 and caspase-8 protein expression was found to be significantly decreased after *C. sativa* treatment at different concentrations in MCF-7 cells, as shown in Figure 8.

Although the MCF-7 and the MDA-MB-231 cell lines have the wild-type and mutant p53, respectively, it is, therefore, important to note that most cancer therapies aim to activate or restore the function of both the wild-type p53 and mutant p53 type, as a functional p53 protein typically acts as a tumor suppressor protein that regulates the cell cycle and promotes apoptosis in response to cellular stress or DNA damage [61,62,63]. Therefore, understanding how wild-type and mutant forms of p53 influence these processes can provide insights into the mechanisms underlying cancer progression and therapy response. In our study, DCM extract increased mutant p53 and the well as expression of p53, and caspase-8 and -9, between the control and treated groups in MDA-MB-231 cells. These results support the hypothesis that the DCM extract of *Cannabis sativa* could inhibit the growth of MDA-MB-231 cells through apoptosis induced by the mitochondrial pathway.

In the current study, we assessed total protein levels of apoptotic markers, including caspase-8 and -9, rather than their cleaved forms. These caspases are synthesized as inactive pro-enzymes. Caspase-2, -8, -9, and -10 are essential for initiating the apoptotic processes, which, once activated, rapidly lead to the cellular changes observed during apoptosis [64]. The Caspase-8 and -9 proteins regulate apoptosis; their activation leads to the cleavage and activation of downstream effector caspases, ultimately resulting in cell death. Due to their dysfunction or reduced activity, they stimulate cell proliferation, malignant transformation, and tumor progression [65].

Indeed, the activation of these caspases through cleavage is a critical step in the apoptotic pathway. In some publications, where the inactive forms have been used, the result is expressed in relation to the untreated sample [66,67,68]. However, to accurately assess apoptotic processes, future studies will focus on evaluating the cleaved forms of these markers to explicitly confirm the observed results.

### 2.8. Phytochemical Screening of Extracts

The phytochemical analysis of DCM extract revealed terpenoids and steroids only (see Table 3); this was because DCM had moderate polarity, and as such, polar compounds like polyphenol were not expected to be present.

**Figure 9:**
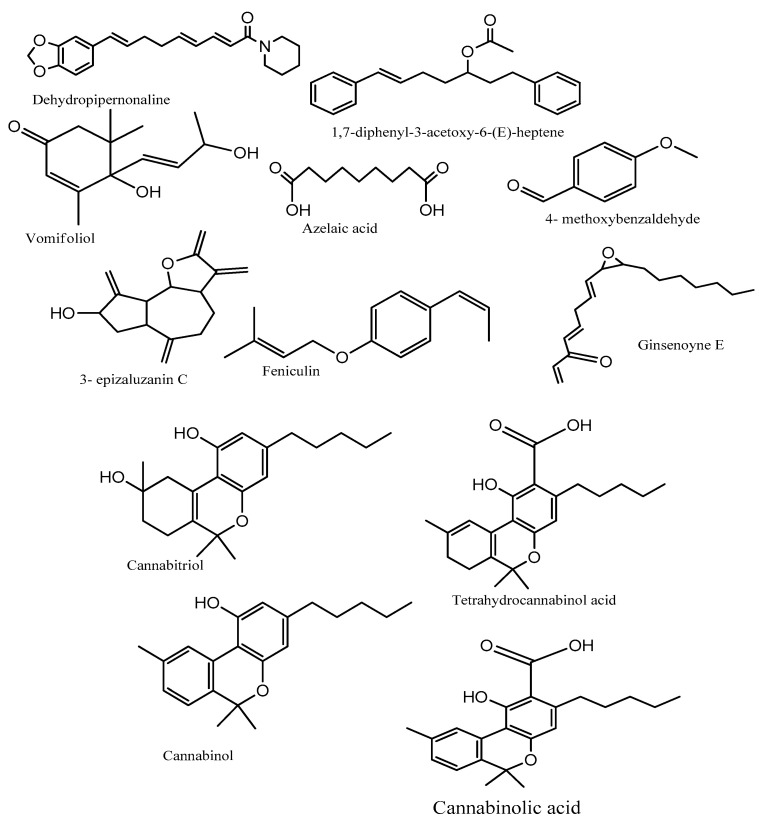
Chemical structures of identified bioactive metabolites in DCM extract.

The UPLC-MS characterization of DCM extract identified cannabinoids: cannabinolic acid with retention time (RT) (12.47 min), cannabidiol (RT. 12.22 min), tetrahydrocannabinol acid (RT. 13.5 min) and terpenoids: vomifoliol (RT. 6.47 min), 3-epizaluzanin C (RT. 9.69 min) and feniculin (RT. 10.47 min) (presented in Table 4, Figure 9 and Figure S1). These identified terpenoids have been reported to possess anti-inflammatory, antioxidant, and anticancer activities [69,70,71]. Further investigation on the presence of cannabinoids in DCM extract using TLC showed the presence of CBD and THCVA, as the extract and standard cannabinoid had the same RF value (Figure S2). The presence of cannabinoids and terpenoids as major bioactive compounds in the DCM extract of *C. sativa* could be responsible for its observed anticancer activity. Other compounds, such as Dehydropipernonaline, are known as pro-oxidants in prostate cancer cells [72]. Our findings corroborate previous reports, in that Phytocannabinoids, derived from *cannabis*, have shown anti-cancer activity in cell lines and demonstrated cytotoxic effects against several cancer types, such as breast, gastric, prostate, lung, liver, oral, and colon cancer cells [30,31,33,34,35,36,37].

The study results indicate that *C. sativa* DCM extract induces the activation of caspase-8 and p53, suggesting that they can trigger the extrinsic apoptotic pathway in breast cancer cells. This is consistent with previous research showing that phytocannabinoids can induce apoptosis through the activation of caspase-8, caspase-9, and p53, and other caspases in various cancer cell lines [64,65]. Many flavonoids exhibit strong antioxidant properties that modulate oxidative stress and scavenge ROS, protecting normal cells while enhancing chemotherapy by increasing cancer cell susceptibility to damage [73]. Identified cannabinoids and terpenoids, such as cannabidiol and tetrahydrocannabinol, modulate oxidative stress by enhancing Superoxide Dismutase (SOD) and Glutathione (GSH) levels, which decreases cellular ROS. This reduction in the Nrf2 pathway promotes pro-oxidant gene expression, leading to increased resilience against oxidative damage. Additionally, these compounds facilitate the activation of caspase-8 and -9, promoting apoptosis in breast cancer cells. By downregulating MMP and TGF-β pathways, cannabinoids and terpenoids also impede invasion and metastasis [58,59,60,74,75].

Thus, the findings of the present study indicated considerable anti-cancer potential of *C. sativa* DCM extract. However, the results need to be further validated in animal models of breast cancer.

## 3. Materials and Methods

### 3.1. Collection and Extraction

*Cannabis sativa* L. aerial parts were collected in the Emampondweni in the Eastern Cape with a permit (number POS 050/2024/2025) from the South African Health Products Regulatory Authority (SAHPRA) for research, storage, possession, and handling of cannabis for research purposes. The plant was authenticated by “Geo Potts Herbarium” at the University of the Free State, Bloemfontein, South Africa. The extractions were performed by 72 h maceration in a mechanical shaker sequentially with n-hexane and Dichloromethane (DCM) at ambient temperature. The filtrates of the extracts were concentrated using a vacuum evaporator. The dried extracts were stored in a cold room (4 °C) until use.

### 3.2. Cytotoxicity in MDA-MB-231 and MCF-7 Breast Cancer Cells

Breast cancer cell lines MDA-MB-231 and MCF-7, along with normal skin fibroblast cells (Hs27), were grown in Dulbecco’s modified Eagle’s medium (DMEM) 30-2002™ supplemented with 10% fetal bovine serum (FBS) (Hyclone, Thermo Fisher Scientific Inc., New York, NY, USA). The cells were maintained at 37 °C in an atmosphere containing 5% CO_2_. Cells were sub-cultured every 2–3 days. After trypsinization, the cell numbers were determined using the Countess II Automated Cell Counter (Thermo Fisher Scientific, Frederick, MD, USA). A cell viability assay was then performed using a 3-(4,5-dimethylthiazol-2-yl)-2,5-diphenyltetrazolium bromide (MTT) assay.

Each well was seeded with 1 × 10^4^ cells in 100 μL of growth medium. The next day, the cells were exposed to different concentrations of extracts (12.5–200 μg/mL), negative control (0.6% DMSO), and doxorubicin (0.1–10 μg/mL) for 24 h, respectively, in a total volume of 200 μL. After incubation, 100 μL of MTT solution (0.5 mg/mL) was added to each microplate well. Then, the plates were again incubated for 2 h in 5% CO_2_ at 37 °C in dark conditions. The formazan crystals created by using MTT-exposed live cells were dissolved by adding 100 μL of dimethyl sulfoxide (DMSO). Three independent experiments were conducted, each in triplicate. Adsorption of the dissolved formazan crystals was measured using a spectrophotometer at 570 nm.

### 3.3. Determination of IC_50_ Values and Selectivity Indexes

The cell viability was calculated as a percentage of the negative control (cells treated with 0.6% DMSO). The percentage of cell viability served as a quantitative indicator of the cellular metabolic activity of live cells, and lower cell viability indicated greater potency of tested samples.

Cell viability (%) = [(A_0_ − A_1_)/(A_2_ − A_1_)] × 100 (1)

A_0_ is the absorbance of the sample, A_1_ is the absorbance of the blank, and A_2_ is the absorbance of the negative control (0.6% DMSO).

The 50% inhibitory concentration (IC_50_) for cancer cells and normal cells was determined with GraphPad Prism software version 8, and a non-linear regression curve. The selectivity index of the extract was calculated using Equation (2).

Selectivity index = IC_50_ normal cells/IC_50_ cancer cells (2)

### 3.4. Clonogenic Assay

One thousand cells were seeded into each well of a 6-well plate and incubated overnight. The following day, each well was treated with varying concentrations of *C. sativa* DCM extract (_1/2_ IC_50_, IC_50_, and 2 × IC_50_) for 24 h. The media was replaced with fresh media every three days, and the plates were incubated for a total of 15 days. After the incubation period, the media were removed, and the cells were washed twice with phosphate-buffered saline (PBS). Cells were then fixed in 2 mL of 100% methanol for 15 min, after which methanol was removed, and samples were allowed to dry at room temperature. Colonies were stained for 30 min using 0.5% crystal violet in methanol. After staining, the plates were rinsed with tap water and allowed to dry. Images of the colonies in each well were captured using a digital camera. Moreover, crystal violet bound to the colonies was dissolved in a solution of 33% acetic acid, and the absorbance was measured at 550 nm [76].

### 3.5. Anti-Metastatic Activities

#### 3.5.1. Cell Migration Assay (Wound Healing Assay)

MDA-MB-231 and MCF-7 cells were seeded in 6-well plates (2 × 10^5^ cells/mL) in the DMEM complete culture medium supplemented with 10% FBS at 37 °C and 5% CO_2_ until confluent. A scratch wound was created by using a sterile 100 μL micropipette tip, then wells were washed with PBS to remove suspended and floating cell debris. The cells were starved with medium with 1% FBS, and incubated with *C. sativa* extract concentrations (1/2 IC_50_, IC_50_, and 2 × IC_50_) were used to treat the MDA-MB-231 and MCF-7 cells, and they were incubated for up to 24 h [77,78]. Cell migration was observed at 0, 12, and 24 h, and images were captured using an inverted microscope equipped with a digital camera. The experiments were conducted in three repetitions (*n* = 3). The width of the scratch and wound closure at different time intervals (0, 12, and 24 h) was analyzed using ImageJ (Js v0.6.0). The relative wound closure was calculated using Equation (3) below. Wound closure was plotted against concentrations of test samples. Two independent experiments were conducted, each in triplicate, and the data are presented as the mean ± standard deviation (SD) with *n* = 3.

Migration % = [(Initial − Final) ÷ Initial] × 100(3)

where Initial = wound surface area at 0 h before migration. Final = wound surface area at a specific time after migration.

#### 3.5.2. Metalloproteinase Inhibitory Activity

MDA-MB-231 and MCF-7 (2.5 × 10^5^ cells/well) were seeded in a 6-well plate and incubated overnight. The medium was removed, and the cells were treated and stimulated for 24 h with *Cannabis sativa* extract in the absence and presence. The supernatants were collected and used for measuring MMP-1, MMP-9, and TGF-beta by ELISA using commercial kits from Invitrogen (Thermo Fisher Scientific, Vienna, Austria) (Human MMP-1, Human MMP-9, and Human TGF-beta 1) according to the manufacturer’s instructions. Each experiment was performed in triplicate. The concentrations were calculated based on the standard curve and then normalized to the control.

### 3.6. Oxidative Activities (Antioxidant Defenses in Cell Line Exposed to Extract or Oxidative Factor Detection)

#### 3.6.1. Superoxide Dismutase (SOD) Activity

SOD activity of *Cannabis sativa* was measured using a SOD colorimetric activity kit (Catalog# CS0009, Sigma-Aldrich, Darmstadt, Germany). Fresh samples (2.5 × 10^5^ cells/mL) were centrifuged at 8000× *g* for 5 min at 4 °C to obtain pellets. These pellets were resuspended with PBS and sonicated for 30 s. Samples were centrifuged at 1500× *g* for 10 min at 4 °C to collect the supernatant. SOD activity assay was performed using the supernatant according to the manufacturer’s instructions. SOD concentration (U/mL) was measured at 450 nm using a microplate spectrophotometer (Thermo Scientific, Multiskan Go).

#### 3.6.2. Glutathione (GSH) Level

GSH level was measured using a glutathione colorimetric detection kit (Catalog # EIAGSHC, Invitrogen™, Frederick, MD, USA). Briefly, fresh samples (2.5 × 10^5^ cells/mL) were centrifuged at 8000× *g* for 5 min at 4 °C to obtain pellets. The samples were homogenized with 5% SSA (aqueous 5-sulfosalicylic acid) in ice-cold PBS and incubated at 4 °C for 10 min. Homogenized samples were then centrifuged at 13,000 rpm for 10 min at 4 °C to collect the supernatant for the GSH assay. The GSH was performed using a glutathione colorimetric detection kit (Catalog # EIAGSHC, Invitrogen™, Frederick, MD, USA) according to the manufacturer’s protocol. Lastly, glutathione activity (U/mL) was measured at 405 nm using a microplate spectrophotometer.

#### 3.6.3. Quantification of Expression of Human Nrf2 in Cancer Cells

The Enzyme-linked Immunosorbent Assay (ELISA) technique was applied to determine the expression levels of Nrf2 after treatment of cancer cells with *Cannabis sativa* DCM extracts. In fact, the cancer cells (2.5 × 10^5^ cells/well) were seeded into 6-well plates and were allowed to attach overnight under standard cell culture conditions. These cells were treated with different concentrations (1/2 IC_50_, IC_50_, and 2 × IC_50_) of *Cannabis sativa* fractions, 0.6% DMSO (negative control). The plates were incubated for 24 h under standard cell culture conditions. After that, cells were washed twice with phosphate-buffered saline (PBS), and the cell lysates were obtained in M-PERTM Mammalian Protein Extraction Reagent containing EDTA-free Pierce Protease and Phosphatase Inhibitor Tablets (Thermo Fisher Scientific, Lenexa, KS, USA). The cell homogenates were centrifuged at 13,000 rpm at 4 °C, and aliquots of cell supernatants were kept at −20 °C. After determining the quantity of protein in each well cell lysate with Pierce TM BCA protein assay kit (Thermo Fisher Scientific, Lenexa, KS, USA), equal amounts of proteins from each cell lysate were used for the quantification of the expression levels of human Nrf2 using the human Nrf2 ELISA Kit according to the manufacturer’s instructions.

### 3.7. Western Blotting Apoptotic Activities

Cells were seeded in 6-well plates at 2.5 × 10^5^ cells per well and followed by overnight incubation before treating with extract at indicated concentrations (1/2 IC_50_, IC_50_, and 2 × IC_50_) for 24 h. Protein lysates were extracted from the control and treated cells. The protein concentration in the supernatants was determined by the BCA protein assay kit. Then, equal amounts of protein were run on 12% Sodium-Dodecyl-Sulfate Poly Acrylamide Gel Electrophoresis (SDS-PAGE) and were electro-transferred into membranes. The membranes were being blocked with 5% *w*/*v* non-fat milk in Phosphate-Buffered Saline (PBS) Tween-20; incubated overnight at 4 °C with primary antibodies, mouse caspase-8, rabbit anti-p53, and rabbit anti-GAPDH; rinsed; and then incubated for 1 h at 25 °C with AP124 Goat anti-Mouse IgG antibody or horseradish peroxidase-conjugated secondary antibodies. The blots were then developed by using the colorimetric method (NBT/BCIP, Thermofisher, Rockford, IL, USA) or Chemiluminescence detection. Densitometry analysis of the protein bands was performed using ImageJ Software.

### 3.8. Phytochemical Analysis

The hexane and dichloromethane extracts were screened for the presence of bioactive metabolites using standard methods of [79].

**UPLC-PDA-HRMS profiling of dichloromethane extract**

The phytochemical analysis of DCM extract was performed on an ACQUITY UPLC (Waters Corporation, Milford, MA, USA) system coupled to a photodiode array detector (PDA) and a Waters^®^ XEVO-G2-XS-QTOF (Waters Corporation, Milford, MA, USA) (UPLC-PDA-HRMS). The ACQUITY UPLC^®^ C18 BEH (1.7 μm pa, 2.1 × 100 mm) analytical column at 50 °C using reverse phase step gradient consisting of water (mobile phase A) and methanol (mobile phase B), each spiked with 0.1% formic acid (FA), was used for the separation. The DCM extract was prepared by dissolving 1mg of extract in 1 mL of 1:1 MeOH/H_2_O, and filtered into UPLC vials using nylon filters (0.22 µm pore size). The gradient elution was for the analysis of DCM extract with solvent mixture ratio water 97% (solvent A) and methanol 3% (solvent B), and water 0% (solvent A) and methanol 100% (solvent B). The elution flow rate was 0.3 μm/mL, and the process started from 1 to 17 min. The sample was analyzed in a single 9.5 µL injection on a QTOF-MS detector in positive and negative ESI mode, and data were acquired using MassLynx v4.1 software (Waters Corporation, Milford, MA, USA).

**TLC profile of DCM extract**

The DCM extract and standard cannabinoids (1 mg) were each dissolved in 1 mL of ethanol and filtered. The filtrates (2 µL) each were applied on a precoated TLC aluminum sheet silica gel 60 with fluorescent indicator (Macherey-Nagel, Düren, Germany). The solvent system used for the prepared plate development was a mixture of petroleum ether and diethyl ether in a 7:3 ratio. The developed plate was air-dried and visualized in a UV chamber at a wavelength of 254 nm.

### 3.9. Statistical Analysis

All experiments were carried out in triplicate (*n* = 3) and presented as the mean ± SD (standard deviation). The statistical analysis of data was performed with GraphPad Prism 8.0.2 software using a one-way analysis of variance (ANOVA) and Dunnett’s test. Results were considered significantly different when *p* < 0.05.

## 4. Conclusions

Based on these research findings, we concluded that *C. sativa* DCM extract possesses the potential to inhibit the proliferation of breast cancer cells (MCF-7 and MDA-MB-231), while exhibiting minimal cytotoxic effect on normal skin cells Hs27. The anti-proliferative effects of *C. sativa* DCM are mainly attributed to the modulation of key molecular pathways, including the inhibition of SOD activity and GSH level, as well as the suppression of MMP-1, MMP-9, and TGF-β in both cancer cell lines. In addition, *C. sativa* DCM promotes apoptosis in these cell lines by increasing the expression of p53, caspase-8 and -9, while suppressing the expression of Nrf2.

Given these conclusive findings, the presence of bioactive phytochemicals in *C. sativa* DCM can be considered as a potential source of anti-cancer agents. To fully validate its therapeutic potential, further empirical research, using in vivo models and clinical studies, is needed to support the results obtained in this study.

## Figures and Tables

**Table 1:** Inhibitory concentration (IC_50_) values of different *Cannabis sativa* extracts against breast cancer and skin fibroblast cells (Hs27).

	MDA-MB-231	MCF-7	Hs27
	IC_50_ (μg/mL)	IC_50_ (μg/mL)	IC_50_ (μg/mL)
DCM	75.46 ± 0.13	78.68 ± 0.50	142.26 ± 2.57
Hex	>200	>200	>200
Doxorubicin	0.1 ± 0.58	0.072 ± 0.36	9.30 ± 0.28

Data are presented as means of triplicate measurements ± standard error of mean. Values are significantly different at *p* < 0.05. All experiments were carried out in triplicate (*n* = 3), and the results are presented as mean ± SEM (standard error of mean) values. IC_50_—concentration required to inhibit the cell growth by 50% compared to negative control (DMSO 0.6%); Hex—hexane extract; DCM—dichloromethane extract; DMSO—dimethylsulfoxide.

**Table 2:** Selectivity index (SI) values of plant extracts as compared to skin fibroblast cells (HS27).

	Selectivity Index	
	MDA-MB-231	MCF-7
DCM	1.88	1.80
Hex	nd	nd
Doxorubicin	93	129.16

nd = not determined; Hex—hexane extract; DCM—dichloromethane extract.

**Table 3:** Bioactive metabolites detected in hexane and DCM extracts.

Bioactive Metabolites	Test	DCM Extract	Hex Extract
Flavonoids	10% NaOH	Absent	Absent
Terpenoids	Liberman Burchard Salkowski	Present	Present
Steroids	Liberman Burchard Salkowski	Present	Absent
Tannins	FeCl_3_	Absent	Absent

Hex—hexane extract; DCM—dichloromethane extract.

**Table 4:** Bioactive metabolites identified in DCM extract.

Peak No.	Name of Compound	R_t_ (Min)	Molar Mass (g/mol)	Formula
1 (+ mode)	1,7-diphenyl-3-acetoxy-6(E)-heptene	10.55	326.2112	C_21_H_24_O_2_
2 (+ mode)	Cannabinolic acid A	12.47	355.1898	C_22_H_26_O_4_
3 (+ mode)	Dehydropipernonaline	13.28	340.1902	C_21_H_25_NO_3_
4 (+ mode)	Unidentified	14.76	149.0231	C_8_H_4_O_3_
1 (− mode)	Vomifoliol	6.47	269.1393	C_13_H_20_O_3_
2 (− mode)	Azelaic acid	7.89	187.0972	C_9_H_16_O_4_
3 (− mode)	4-Methoxybenzaldehyde	8.50	181.0497	C_8_H_8_O_2_
4 (− mode)	3-Epizaluzanin C	9.69	245.1183	C_15_H_18_O_3_
5 (− mode)	Feniculin	10.47	247.1331	C_14_H_18_O
6 (− mode)	Ginsenoyne E	11.05	317.1751	C_17_H_22_O_2_
7 (− mode)	Cannabidiol	12.22	345.2056	C_21_H_30_O_4_
8 (− mode)	Tetrahydrocannabinol acid	13.15	357.2065	C_22_H_30_O_4_
9 (− mode)	Cannabinol	13.60	309.1861	C_21_H_26_O_2_
10 (− mode)	Cannabinolic acid A	14.34	353.1758	C_22_H_26_O_4_

R_t_—retention time.

## Data Availability

The original contributions presented in this study are included in this article/Supplementary Materials. Further inquiries can be directed to the corresponding authors.

## Supplementary Materials

The following supporting information can be downloaded at: https://www.mdpi.com/article/10.3390/ijms27010152/s1.

## Abbreviations

SOD	Superoxide dismutase
GSH	Glutathione
DCM	Dichloromethane extract
p53	Tumor suppressor protein 53
Nrf2	Nuclear factor erythroid 2-related factor 2
MMP	Metalloproteinase
TGF-beta 1	Transforming growth factor beta1
