# Supplementary material for: Potential Anticancer Effect of Cannabis sativa L. Dichloromethane Extract Through Oxidative Stress-Related Pathways and the Inhibition of the Migration and Invasiveness of Human Breast Cancer Cells (MDA-MB-231 and MCF-7)
Source: Int J Mol Sci. 2025 Dec 23;27(1):152. doi: 10.3390/ijms27010152 (PMC12785486; doi:10.3390/ijms27010152)
Supplement: Supplementary file 1 [file ijms-27-00152-s001.zip › Figure S1.pdf]

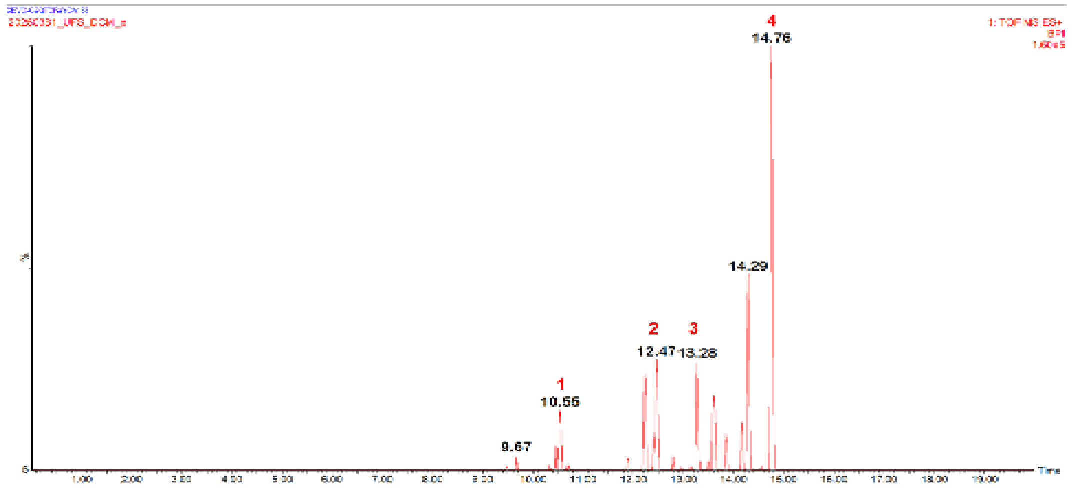

UPLC-MS positive mode

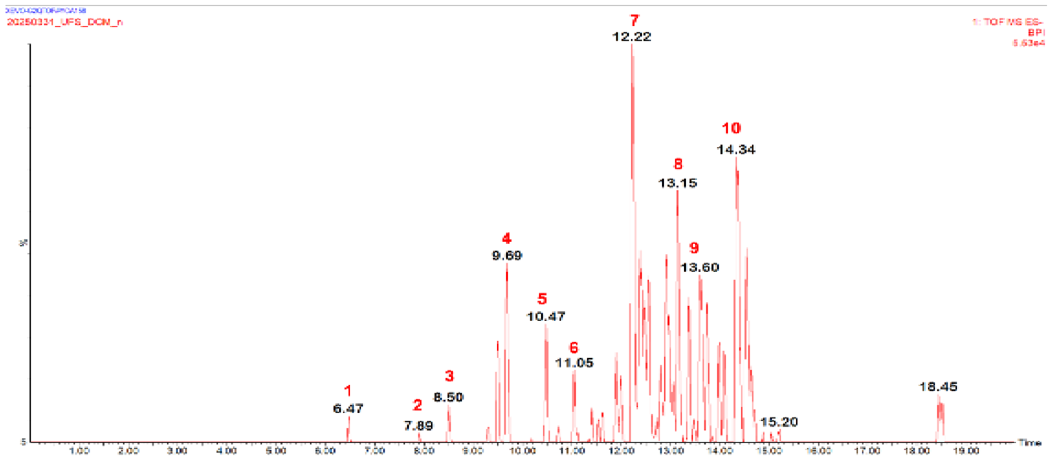

UPLC-MS negative mode

Figure S1: Ultra performance liquid chromatography (UPLC-PDA-HRMS) of DCM extract in both, negative and positive modes. Number compounds in table 4
