# Supplementary figures and images for: Potential Anticancer Effect of Cannabis sativa L. Dichloromethane Extract Through Oxidative Stress-Related Pathways and the Inhibition of the Migration and Invasiveness of Human Breast Cancer Cells (MDA-MB-231 and MCF-7)
Source: Int J Mol Sci. 2025 Dec 23;27(1):152. doi: 10.3390/ijms27010152 (PMC12785486; doi:10.3390/ijms27010152)

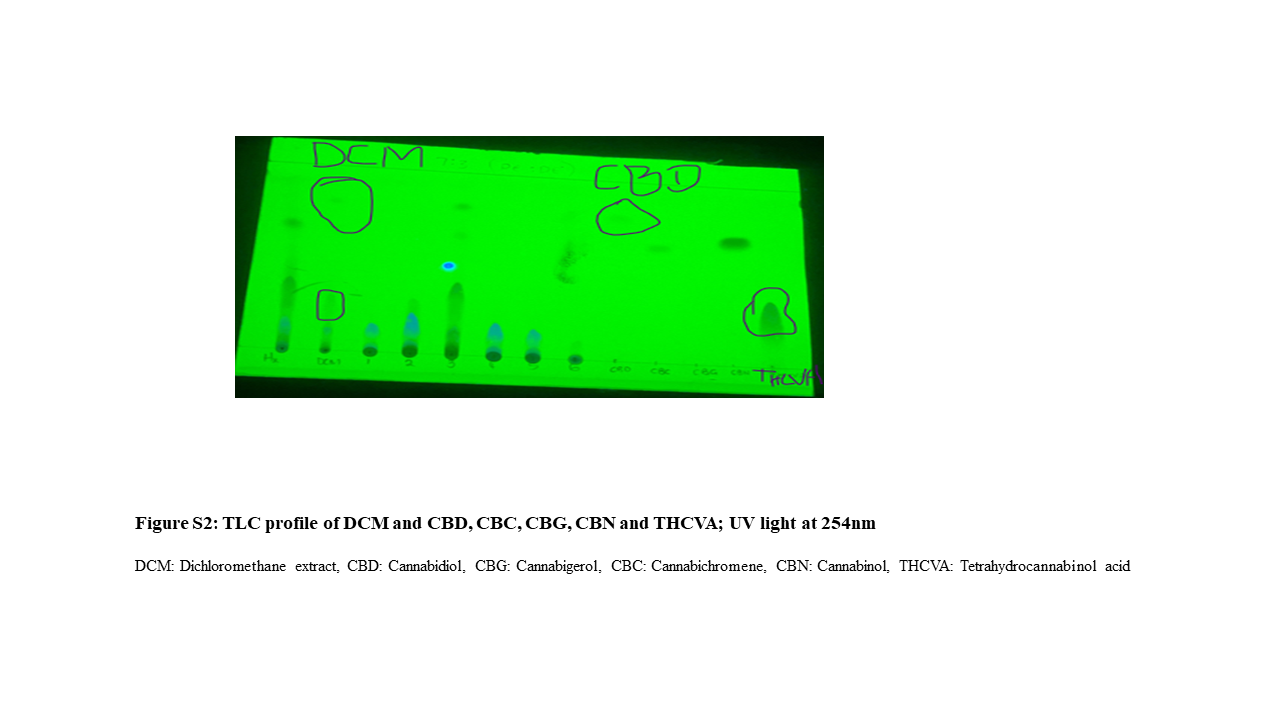

Supplement: Supplementary file 1 [file ijms-27-00152-s001.zip › Figure S2.tif]
